# Responsiveness: a reinvention of the wheel?

**DOI:** 10.1186/1477-7525-3-8

**Published:** 2005-02-03

**Authors:** Robert Lindeboom, Mirjam A Sprangers, Aeilko H Zwinderman

**Affiliations:** 1 Departments of Clinical Epidemiology and Biostatistics, Academic Medical Center, Amsterdam, the Netherlands; 2 Medical Psychology, Academic Medical Center, Amsterdam, the Netherlands

**Keywords:** quality of life, questionnaires, effect size, psychometrics, scale evaluation, reliability

## Abstract

**Background:**

Since the mid eighties, responsiveness is considered to be a separate property of health status questionnaires distinct from reliability and validity. The aim of the study was to assess the strength of the relationship between internal consistency reliability, referring to an instrument's sensitivity to differences in health status among subjects at one point in time, and responsiveness referring to sensitivity to health status changes over time.

**Methods:**

We used three different datasets comprising the scores of patients on the Barthel, the SIP and the GO-QoL instruments at two points in time. The internal consistency was reduced stepwise by removing the item that contributed most to a scale's reliability. We calculated the responsiveness expressed by the Standardized Response Mean (SRM) on each set of remaining items. The strength of the relationship between the thus obtained internal consistency coefficients and SRMs was quantified by Spearman rank correlation coefficients.

**Results:**

Strong to perfect correlations (0.90 – 1.00) was found between internal consistency coefficients and SRMs for all instruments indicating, that the two can be used interchangeably.

**Conclusion:**

The results contradict the conviction that responsiveness is a separate psychometric property. The internal consistency coefficient adequately reflects an instrument's potential sensitivity to changes over time.

## Background

Responsiveness, a concept introduced in the mid-eighties by bio-medical researchers, is considered to be an essential measurement property of health status questionnaires, distinct from reliability and validity [1]. However, it can be questioned whether an instrument's sensitivity to differences between health status changes over time, which refers to responsiveness, is different from an instrument's sensitivity to differences in health among subjects at one point in time, which refers to the psychometric concept of parallel forms reliability from the framework of classical test theory [2]. A number of theorists have argued that responsiveness is not a separate psychometric attribute of health status instruments, but merely some form of construct validity [3]. The aim of the study is to provide empirical evidence of this notion by investigating the relationship between instrument responsiveness and the traditional psychometric concept of parallel forms reliability as embodied by the internal consistency coefficient [2,3].

## Methods

We used three datasets comprising the scores of patients on three widely used health status instruments on two moments in time in order to assess health changes. The first dataset was from a randomized clinical trial investigating the effects of arm and leg rehabilitation training on the functional recovery of stroke survivors using the 10-item Barthel (basic) activities of daily living scale [4]. Patients (n = 89) were rated one week and 12 weeks after stroke. A Barthel scale score ranges from zero to 20 points with higher scores indicating more independent functioning. The second dataset comprised the scores on the 45-item physical component of the Sickness Impact Profile (SIP) of 227 patients with myocardial infarction [5](on average 2 year interval between assessments), 120 patients with stroke [6] (3 year interval between assessments) and 141 patients scheduled for a carotid endartectomy surgical procedure [7] (3 months time interval). The SIP physical items are scored on a dichotomous scale with 1 point for each endorsed item statement. The scale ranges from 0 to 45 points with higher scores indicating higher levels of sickness related dysfunction. The third dataset contained the scores of 164 patients with Graves' ophthalmopathy scored on the 8-item psychosocial dimension of the Graves' ophthalmopathy quality of life (GO-QOL) instrument [8]. The GO-QOL scale items are scored on a 1 to 3 point rating scale. Overall scores are transformed to a 0–100 scale with higher scores indicating better psychosocial functioning. Patients completed the instrument before and three or six months after radiotherapy or eye surgery.

Only subjects with no missing values at baseline or follow-up were included in the analysis. The Barthel dataset had no missing values, the SIP datasets had 13 % (16/120), 28% (64/227) and 0.7% (1/141) missing values respectively and the GO-QOL had 0.6% (1/164) missing values. For the SIP datasets, there was a mean deterioration in health (1 point), for the Barthel and GO-QOL scales patients were improved at follow-up (Table 1).

**Table 1:** Score statistics and reliabilities (α) at baseline and follow-up.

	Barthel N = 89	SIP N = 407	GO-QOL N = 163
Baseline score (SD, IQR^1^)	7.98 (4.52, 5–12)	5.12 (6.28, 1–7)	59.32 (24.64, 44–81)
Follow-up score (SD, IQR)	14.25 (5.06, 10–19)	6.13 (7.57, 0–9)	65.22 (24.17, 50–81)
Mean change score (SD)^2^	6.27 (3.21)	1.01 (4.36)	5.90 (17.13)
SRM^3^	1.95	0.23	0.34
Chronbach's α time 1	0.86	0.92	0.83
Chronbach's α time 2	0.89	0.94	0.84

1) Distribution of change scores was approximately normal for all three datasets

2) IQR = interquartile range

3) SRM = Standardized Response Mean (see statistical analysis section)

### Statistical analysis

The analysis aimed to assess the strength of the relationship between internal consistency reliability (Cronbach α or Kuder-Richarson-20) reflecting sensitivity to differences in health status among patients [3], and the Standardized Response Mean effect size (SRM) indicating an instrument's sensitivity to change. The SRM is calculated as the mean of the change scores divided by the standard deviation of the change scores [3,9]. In a stepwise procedure, we reduced the baseline internal consistency by removing the item contributing most to the internal consistency coefficient until 0.60 was reached, which was considered as the minimum standard for reliability [10]. For the 45-item SIP physical scale, two items were removed at every step. For the other instruments, one item was removed at each step. Using the remaining items at each item reduction step, we calculated the SRM. The thus decreasing internal consistency coefficients and associated SRMs were plotted and the strength of the relationship was calculated using Spearman rank correlation coefficients. All analyses were performed with SPSS 11.0, a commercially available software package.

## Results

**Figure 1:**
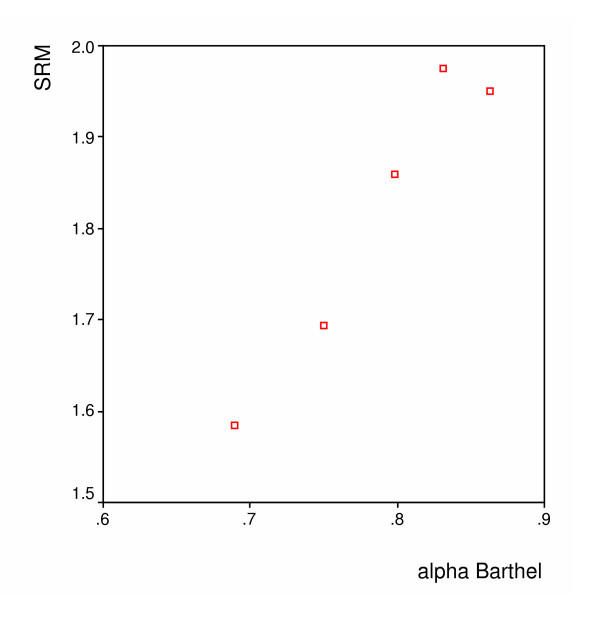
**Barthel – Relation between the internal consistency reliability (alpha) and the SRM **(Spearman's r = 0.90)

**Figure 2:**
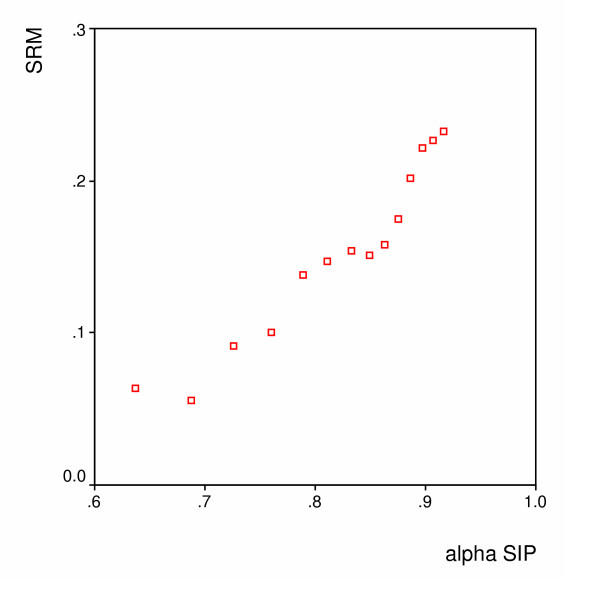
**SIP – Relation between the internal consistency reliability (alpha) and the SRM **(Spearman's r = 0.99)

**Figure 3:**
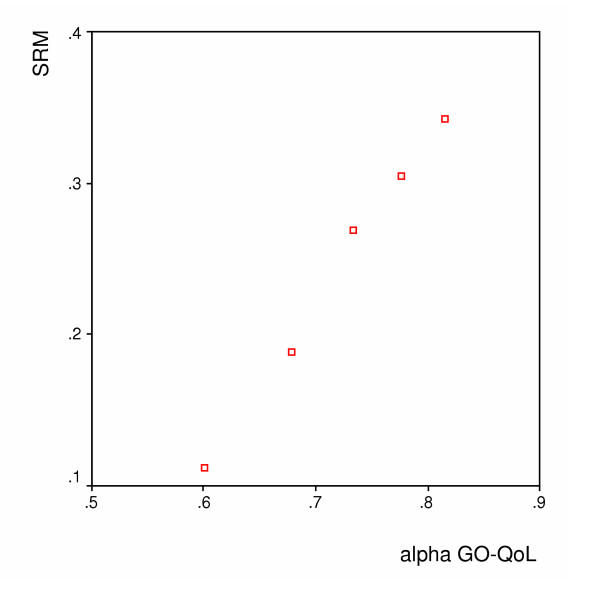
**GO-QOL – Relation between the internal consistency reliability (alpha) and the SRM **(Spearman's r = 1.00)

Figures 1, 2 and 3 show the spearman rank correlations between the internal consistency coefficients and the SRM using the scores on the Barthel, the SIP and the GO-QOL respectively. The spearman rank correlations ranged between 0.90 for the Barthel index to 1.00 for the GO-QoL indicating strong to perfect relations between internal consistency and responsiveness.

## Discussion

Our results contradict the conviction that responsiveness is a separate psychometric property of health scales. Internal consistency reliability, reflecting a scale's sensitivity to cross-sectional differences in health, closely coincided with the instruments' sensitivity to change as measured with the standardized response mean. Our results also reflect what is already known within the framework of classical test theory. A test score cannot correlate more highly with any other variable than its own true score [2]. This implies that the maximum correlation between an observed test score and any other variable, i.e. its validity, is the square root of its reliability [2]. Thus, the more reliable a test, the more potential for validity, in this case responsiveness, there exists. We used nested versions of the same test, which are highly correlated with each other, to illustrate this phenomenon. It is likely, however, that the results will also apply with different instruments measuring similar health constructs that are highly inter-correlated. It should also be noted that the results apply to one-dimensional psychometric scales and not to instruments containing so-called "causal" variables, for example disease symptoms [3] since these instruments are not strictly one-dimensional.

We used the SRM effect size that uses the standard deviation the change scores and therefore includes all information about the changes on the selected instruments. The results can not generalized to alternative effect sizes such as Cohen's effect size or Guyatt's responsiveness statistic [1] because these largely depend on the variability of scores at baseline or the variability in scores obtained from a separate, not improved, sample.

In a frequently cited paper, Guyatt et al. [1] made the distinction between discriminative instruments, whose purpose it is to measure differences between subjects and evaluative instruments, designed to examine change over time. This in contrast to most of the scales used in clinical medicine (blood pressure, cardiac output), which are assumed to work well in both discriminative and evaluative roles. To corroborate his arguments, he used the hypothetical example of two health status instruments designed to evaluate therapeutic interventions in patients with chronic lung disease that were presented to the same patient sample (Table 2). "Evaluative" instrument A showing poor test-retest reliability because of small between subject score variability but excellent responsiveness, and "discriminative" instrument B with excellent reliability because of large between-subject score variability and poor responsiveness. From Table 2, however, it can be seen that this representation of instrument behaviour in clinical research is logically inconsistent, since it does not explain how two instruments, both measuring the same health construct show such divergent score distributions at baseline. According to instrument A the sample is highly homogeneous, while it is highly heterogeneous according to instrument B. In Appendix 1 (see additional file 1), we show that the above representation is not impossible, but highly unlikely since it occurs only in extreme situations.

**Table 2:** Representation of the scores on "evaluative" instrument A and "discriminative" instrument B in a randomized clinical trial [1]

Instrument A	Time 1	Time 2	Intervention	Time 3	Difference score	Exercise test Result
Subject 1	8	9	Verum	15	+6	Much improved
Subject 2	9	8	“”	15	+7	Much improved
Subject 3	8	9	“”	15	+6	Much improved
Subject 4	9	8	“”	15	+7	Much improved
Subject 5	8	9	Placebo	8	-1	Unchanged
Subject 6	9	8	“”	9	+1	Unchanged
Subject 7	8	9	“”	8	-1	Unchanged
Subject 8	9	8	“”	9	+1	Unchanged
						
Instrument B	Time 1	Time 2		Time 3	Difference score	Exercise test Result

Subject 1	5	5	Verum	5	0	Much improved
Subject 2	9	9	“”	9	0	Much improved
Subject 3	13	13	“”	13	0	Much improved
Subject 4	17	17	“”	17	0	Much improved
Subject 5	5	5	Placebo	5	0	Unchanged
Subject 6	9	9	“”	9	0	Unchanged
Subject 7	13	13	“”	13	0	Unchanged
Subject 8	17	17	“”	17	0	

During the past 20 years, clinimetric research has resulted in about 25 definitions and 30 measures of instrument responsiveness, sometimes referred to as sensitivity to change or longitudinal validity [11]. Moreover, it is evaluated in literally hundreds of published papers on the validation of health status instruments. Our results show that responsiveness, as measured with the SRM, mirrors the traditional concept of parallel test reliability as embodied by the internal consistency coefficient. When comparing instruments measuring similar health constructs, an instrument sensitive to health differences among subjects is also likely to be sensitive to therapy-induced change as well. However, further empirical data will be needed to confirm the relationship between internal consistency and responsiveness, e.g., by reviewing studies in which health status instruments were compared on their responsiveness.

## Authors' contributions

Robert Lindeboom conceived the idea for the manuscript, Mirjam Sprangers and Robert Lindeboom wrote the manuscript, Koos Zwinderman provided the mathematics and rewrote parts of the manuscript in earlier drafts

## Supplementary Material

Additional File 1 Appendix 1: Is it possible to have one high reliable scale with low 'responsiveness', and a low reliable scale with high 'responsiveness' measuring the same construct? Click here for file
