# Supplementary material for: Responsiveness: a reinvention of the wheel?
Source: Health Qual Life Outcomes. 2005 Feb 3;3:8. doi: 10.1186/1477-7525-3-8 (PMC549031; doi:10.1186/1477-7525-3-8)
Supplement: Additional File 1 — Appendix 1: Is it possible to have one high reliable scale with low 'responsiveness', and a low reliable scale with high 'responsiveness' measuring the same construct? [file 1477-7525-3-8-S1.doc]

**Appendix 1: Is it possible to have one high reliable scale with low ‘responsiveness’, and a low reliable scale with high ‘responsiveness’ measuring the same construct?**

Consider two scales A and B measuring the same construct θ before and after an effective intervention. Before intervention θ has expectation μ_1_ and variance$\sigma_{1}^{2}$. After the intervention θ has expectation μ_2_ and variance$\sigma_{2}^{2}$. The covariance between values before and after intervention is denoted as σ_12_.

Theta (θ) is a latent construct and is quantified through scales A and B, assuming that both scales conform to the classical test theory. This means that observed values with scale A and B at baseline (Y_1,A_ and Y_1,B_) may be seen as Y_1,A_= θ_1_+e_1,A_ and Y_1,B_= θ_1_+e_1,B_, where e_1,A_ and e_1,B_ denote measurement-error each with expectation zero, and variances$\tau_{1,A}^{2}$ and$\tau_{1,B}^{2}$. Reliability of scales A and B at baseline equals$\sigma_{1}^{2}/(\sigma_{1}^{2}+\tau_{1,A}^{2})$, and$\sigma_{1}^{2}/(\sigma_{1}^{2}+\tau_{1,B}^{2})$, and obviously, if B is more reliable than A, this means that measurement-error of A varies more than that of B: $\tau_{1,B}^{2}$ < $\tau_{1,A}^{2}$. After intervention, classical test theory applies too: Y_2,A_= θ_2_+e_2,A_ and Y_2,B_= θ_2_+e_2,B_, with error-variances $\tau_{2,A}^{2}$ and$\tau_{2,B}^{2}$.

Consider now the standardized response mean (SRM) as a measure of responsiveness. SRM for scale A is defined as

,

where $\bar{d}$is the average change, and s_d_ the standard deviation of the changes, and where

$\bar{y}_{1,A}$ and $\bar{y}_{2,A}$ are the observed means of scale A before and after intervention, and $s_{1,A}^{2}$ and $s_{2,A}^{2}$ the observed variances of scale A before and after intervention, and s_12,A_ the covariance between the observed scale-scores. Obviously, expectation of the numerator of the SRM_A_ equals μ_1_-μ_2_, and the same is the case for the numerator of SRM_B_. Hence, any difference between SRM_A_ and SRM_B_ must be due to their denominators. The expected variances $s_{1,A}^{2}$and $s_{2,A}^{2}$ are, off course, equal to $s_{1,A}^{2}$=$\sigma_{1}^{2}$+$\tau_{1,A}^{2}$, and $s_{2,A}^{2}$=$\sigma_{2}^{2}$+$\tau_{2,A}^{2}$. The expected covariance s_12,A_ equals S_12,A_= σ_12_ + cov(θ_1_,e_1,A_) + cov(θ_2_,e_2,A_) + cov(e_1,A_,e_2,A_). Since e_1,A_ and e_2,A_ are measurement-error terms (before and after intervention), they will have zero covariance, both with θ and with each other. Hence, the denominator of SRM_A_ equals ($\sigma_{1}^{2}$+$\sigma_{2}^{2}$-2σ_12_)+$\tau_{1,A}^{2}$+$\tau_{2,A}^{2}$, and using similar arguments the denominator of SRM_B_ equals ($\sigma_{1}^{2}$+$\sigma_{2}^{2}$-2σ_12_)+$\tau_{1,B}^{2}$+$\tau_{2,B}^{2}$.

Inspection of the denominators of SRM_A_ and SRM_B_ shows that SRM_A_ can only be larger than SRM_B_, when the measurement-error variance of scale A after intervention is smaller than that of scale B:$\tau_{2,A}^{2}$ << $\tau_{2,B}^{2}$. Although this is not impossible, the occurrence is unlikely, especially when the reverse is the case before intervention. It is clear moreover that the difference in responsiveness is only a reflection of a difference in reliability between the two scales, since $\tau_{2,A}^{2}$ << $\tau_{2,B}^{2}$ implies that the reliability of scale B after intervention is lower than of scale A.
